# Antibiogram Signatures of Some Enterobacteria Recovered from Irrigation Water and Agricultural Soil in two District Municipalities of South Africa

**DOI:** 10.3390/microorganisms8081206

**Published:** 2020-08-07

**Authors:** Chidozie Declan Iwu, Erika M du Plessis, Lise Korsten, Nolonwabo Nontongana, Anthony Ifeanyi Okoh

**Affiliations:** 1 SAMRC Microbial Water Quality Monitoring Centre, University of Fort Hare, Alice 5700, South Africa; nnontongana@ufh.ac.za (N.N.); aokoh@ufh.ac.za (A.I.O.); 2 Applied and Environmental Microbiology Research Group, Department of Biochemistry and Microbiology, University of Fort Hare, Alice 5700, South Africa; 3 Department of Plant and Soil Sciences, Faculty of Natural and Agricultural Sciences, University of Pretoria, Pretoria 0002, South Africa; erika.duplessis@up.ac.za (E.M.d.P.); lise.korsten@up.ac.za (L.K.)

**Keywords:** antibiotic resistance, irrigation water, agricultural soil, public health, food safety, food microbiology, environmental health

## Abstract

This study was undertaken to evaluate the antibiogram fingerprints of some Enterobacteria recovered from irrigation water and agricultural soil in two District Municipalities of the Eastern Cape Province, South Africa using standard culture-based and molecular methods. The prevalent resistance patterns in the isolates follow the order: *Salmonella enterica* serovar Typhimurium [tetracycline (92.3%), ampicillin (69.2%)]; *Enterobacter cloacae* [amoxicillin/clavulanic acid (77.6%), ampicillin (84.5%), cefuroxime (81.0%), nitrofurantoin (81%), and tetracycline (80.3%)]; *Klebsiella pneumoniae* [amoxicillin/clavulanic acid (80.6%), ampicillin (88.9%), and cefuroxime (61.1%)]; and *Klebsiella oxytoca* [chloramphenicol (52.4%), amoxicillin/clavulanic acid (61.9%), ampicillin (61.9%), and nitrofurantoin (61.9%)]. Antibiotic resistance genes detected include *tet*C (86%), *sul*II (86%), and *bla*_AmpC_ (29%) in *Salmonella enterica* serovar Typhimurium., *tet*A (23%), *tet*B (23%), *tet*C (12%), *sul*I (54%), *sul*II (54%), *cat*II (71%), *bla*_AmpC_ (86%), *bla*_TEM_ (43%), and *bla*_PER_ (17%) in *Enterobacter cloacae*., *tet*A (20%), *tet*C (20%), *tet*D (10%), *sul*I (9%), *sul*II (18%), FOX (11%) and CIT (11%)-type plasmid-mediated *AmpC, bla*_TEM_ (11%), and *bla*_SHV_ (5%) in *Klebsiella pneumoniae* and *bla*_AmpC_ (18%) in *Klebsiella oxytoca*. Our findings document the occurrence of some antibiotic-resistant Enterobacteria in irrigation water and agricultural soil in Amathole and Chris Hani District Municipalities, Eastern Cape Province of South Africa, thus serving as a potential threat to food safety.

## 1. Introduction

Enterobacteriales is an order made up of diverse groups of opportunistic, non-sporulating, fastidious, Gram-negative facultative anaerobes with the ability to break down sugars to numerous end-products including acids and gas [1,2]. The order includes a number of foodborne pathogens including *Salmonella* spp., *Shigella* spp., *Enterobacter* spp., *Klebsiella* spp. among others, and are generally referred to as “enterics” because of their ability to cause gastrointestinal tract (GIT) infections in humans, especially in the developing parts of the world [3]. The primary habitat for members of this family is the human and animal intestinal tract, and they are usually disseminated via contaminated food and water resources, causing severe healthcare-associated infections [4,5,6] including bloodstream infections, urinary tract infections (UTIs), peritonitis, cholangitis, healthcare-associated pneumonia, and other intra-abdominal infections [7]. They are also implicated in chronic systemic infections such as hemolytic uremic syndrome (*E. coli* O157: H7), acute non-typhoidal infections (*Salmonella* spp.), and severe forms of diarrhea (*Salmonella* spp, *Shigella* spp. and pathogenic *E. coli*) [8].

Members of Enterobacteriales are ubiquitous in nature [9]. They have the ability to survive for extended periods of time in the soil depending on the moisture content, temperature, and soil type [10,11]. Microbial assessments of the agricultural environment have detected members of Enterobacteriales within the various niches of the agro-ecosystem. In the U.S., *Salmonella* spp. was detected from irrigation water, soil, and mid-Atlantic pond sediments found around agricultural farms located within major produce growing regions [12]. The pathogen was also recovered from soil and surface water bodies in New York [13], and also from the soil, irrigation water, and sediment in California [14]. *E. coli* was recovered from various river water used for irrigation of vegetables in South Africa [15,16], while *Salmonella enterica* and Shiga toxin-producing *E. coli* (STEC) was recovered from freshwater sources used for irrigation [13] as well as from watersheds close to vegetable production area in the California central coast agricultural region [17]. This suggests that irrigation water and soil are important reservoirs of Enterobacteria which consequently drives their dissemination to fresh produce [18].

Fresh produce including vegetables and fruits are categorized as “ready-to-eat-foods” because they are either eaten raw or minimally processed before they are finally consumed, resulting in potential food safety issues [19]. Consumption of these food products signifies an integral part of a healthy diet, unfortunately, their contamination with foodborne pathogens poses serious public health consequences [20]. Fresh produce normally harbors non-pathogenic epiphytic organisms, however, contamination of these produce by foodborne pathogens usually occur on the farm through various sources including animal manure, soil, and irrigation water [21,22,23]. This could either occur through surface contamination of the plants by splashes of irrigation water, rain, surface runoffs, soil particles, or through root internalization and natural openings such as stomata or plant injuries [24]. The frequent occurrence of foodborne pathogens including Enterobacteria on commercial agricultural food products contributes to their incorporation into the food web [25], which is clear from the rise in the number of foodborne illnesses and outbreaks related to ingestion of raw ready-to-eat produce such as leafy greens, tomatoes, cabbage, jalapeños, and melons [26,27].

Several Enterobacteria found on fresh produce harbors multidrug resistance determinants, thereby exposing consumers to more safety issues [28,29]. Usually, antimicrobial-resistant pathogens emerging due to the indiscriminate use of antimicrobial agents in both veterinary and agricultural niches escape from the farm environments to the food chain through animal manure, soil, and irrigation water with the potential of transfer of the resistance determinants to other related and non-related species via horizontal gene transfer [18,30,31,32]. The spread of multidrug-resistant Enterobacteria is causing serious challenges to the management of infectious diseases due to the rapid emergence of strains capable of resisting the effects of most currently available antimicrobial agents [33,34,35]. Only a few studies have accessed the occurrence of multidrug-resistant members of Enterobacteriales in irrigation water and agricultural soil. In this paper, we report on the antibiogram signatures of key members of the Enterobacteriales recovered from irrigation water and agricultural soil in Amathole and Chris Hani District Municipalities, Eastern Cape Province of South Africa, as a part of our overall investigation of the reservoirs of antimicrobial resistance determinants in the environment. To the best of our knowledge, this is the first report on this subject in the Eastern Cape Province of South Africa.

## 2. Materials and Methods

### 2.1. Study Area

This study was carried out in Amathole and Chris Hani District Municipalities in the Eastern Cape Province, South Africa. The population size of Amathole and Chris Hani District Municipalities is approximately 880,790 [36] and 840,055 [37], respectively. These District Municipalities are mainly agrarian and provide huge opportunities for investment in agro-processing, particularly due to their proximities to East London and Port Elizabeth ports. Irrigation water and agricultural soil samples were collected from 19 sampling sites located within these District Municipalities. Sites 1 to 14 are in Amathole District Municipality while sites 15 to 19 are in Chris Hani District Municipality as shown in Supplementary Table S1.

### 2.2. Collection of Samples and Isolation of Target Pathogens

Nineteen irrigation water samples (225 mL each) were collected in triplicates per sample site in sterile 1 L sample bottles, while 13 agricultural soil samples (25 g) were collected in triplicates from each of the sampling sites located within the study areas. All the samples were collected on a once-off basis between June and September 2018 and were transported on ice to the laboratory for processing within hours of collection.

On getting to the laboratory, 10 mL of irrigation water samples and 10 g of soil were pre-enriched in 90 mL of Trypticase soy broth (TSB) (Merck, Modderfontein, South Africa) for the enrichment of members of Enterobacteriales including *Enterobacter* spp. and *Klebsiella* spp. After 24 h of aerobic incubation at 37 °C, 1 mL of the TSB culture was transferred into 9 mL of Rappaport Vassiliadis broth (RVB) (Merck, Modderfontein, South Africa) for the enrichment of *Salmonella* spp., and incubated at 48 °C for 24 h. Thereafter, a loop full of all the broth cultures were streaked on various media selective for the isolation of target members of the Enterobacteriales. For the isolation of *Salmonella* spp., a loop full of the RVB culture was streaked on Xylose lysine deoxycholate (XLD) (Merck, Modderfontein, South Africa) agar, while a loop full of the TSB culture was concurrently streaked on Eosin methylene blue (EMB) (Merck, Modderfontein, South Africa) agar and McConkey agar (Merck, Modderfontein, South Africa) for the isolation of *Enterobacter* spp., and *Klebsiella* spp., respectively. Distinct colonies with a transparent zone of a reddish color and a black center on XLD agar, pink to purple colonies with no green metallic sheen on EMB and pink colonies on MacConkey agar were presumptive for *Salmonella* spp., *Enterobacter* spp., and *Klebsiella* spp., respectively. A total of 520 presumptive Enterobacteria including 160 *Salmonella* spp., 180 *Enterobacter* spp., and 180 *Klebsiella* spp. were isolated and purified on nutrient agar and stocked in 25% glycerol at −80 °C for further analyses.

### 2.3. Characterization of Target Pathogens

#### 2.3.1. DNA Extraction

Upon resuscitation of the glycerol stocks using nutrient broth (Merck, Modderfontein, South Africa) and incubation for 24 h at 37 °C, the DNA of the 520 presumptive Enterobacterial isolates were extracted using the boiling technique [38,39] with slight modifications. Briefly, the pure overnight colony on nutrient agar were picked and suspended in 200 µL nuclease-free water contained in sterile Eppendorf tubes and vortexed. This was heated at 100 °C for 10 min using MS2 Dri-Block DB.2A (Techne, Marshalltown, South Africa) to lyse the cells after which they were placed on ice to cool. Thereafter, the lysed cells were centrifuged for 5 min at 15,000 rpm using the PRISMR Centrifuge (Labnet International, Inc, Edison, NJ, USA) and 10 µL of the supernatant which contains the DNA template was used for the PCR assays.

#### 2.3.2. PCR Delineation of the Presumptive Enterobacterial Isolates

Primers used in this study were synthesized by Inqaba Biotechnical Industries (Pty) Ltd., Pretoria, South Africa for the delineation of the presumptive Enterobacteria into clinically relevant members including *Salmonella enterica* serovar Typhimurium (*S.* Typhimurium), *Enterobacter cloacae* (*E. cloacae*), *Klebsiella pneumoniae* (*K. pneumoniae*), and *Klebsiella oxytoca* (*K. oxytoca*). Details of all the primer sequences, target genes, expected amplicon size, and cycling conditions used for the PCR screening are shown in Supplementary Table S2. Each PCR reaction mixture was constituted in a final reaction mixture of 25 µL made up of 12.5 µL PCR master mix (Thermo Scientific, Vilnius, (EU) Lithuania), 0.5 µL each of forward and reverse primers, 6.5 µL of PCR grade water, and 5 µL of DNA template. All the amplifications were done using a MyCyclerTM thermal Cycler PCR system (BioRad, Hercules, CA, USA), and 5 µL of the amplicons thereafter were resolved in 1.8% agarose gel (Separations, Johannesburg, South Africa) stained with ethidium bromide (0.001µg/mL) using 0.5X Tris-borate EDTA (TBE) buffer including a 100-bp DNA ladder (Promega, Madison, WI, USA) which served as a molecular size standard. The electrophoresis was run at 100 V for 60 min and the gels were visualized under the UV trans-illuminator (Alliance 4.7, UVItec, Merton, London, UK). *S.* Typhimurium (DSMZ 14028, DSMZ, Braunschweig, Germany), *E. cloacae* ATCC 13047 (ATCC, Manassas, VA, USA), *K. pneumoniae* ATCC 35657 (ATCC, Manassas, VA, USA), and *K. oxytoca* ATCC 13182 (ATCC, Manassas, VA, USA) were used as reference strains for the PCR confirmation of target isolates.

### 2.4. Antibiotic Susceptibility Testing of Confirmed Members of Enterobacteriales

The confirmed Enterobacteria were subjected to antibiotic susceptibility testing using the disk diffusion test method as recommended by the Clinical Laboratory Standard Institute (CSLI) [40]. Confirmed colonies were picked from 24 h pure culture on nutrient agar, suspended in sterile normal saline, and adjusted to match the 0.5 McFarland standard. The mixture was evenly inoculated on Mueller-Hinton agar using a sterile swab and test antimicrobial agents placed on the agar using a disc dispenser. The plates were incubated for 16 to 20 h at 37 °C. All the inhibition zones were measured in mm and interpreted as Susceptible (S), Intermediate (I), or Resistant (R), or using the standards recommended by the CLSI [40]. A panel of 16 antimicrobial agents belonging to 10 classes and of human and veterinary importance were used in the tests and they include, the aminoglycosides [gentamycin (GM-10 µg), amikacin (AK-30 µg)], β-lactams [amoxicillin-clavulanic acid (AUG-30 µg), ampicillin (AP-10 µg)], carbapenems [imipenem (IMI-10 µg), meropenem (MEM-10 µg)], cephems [cefotaxime (CTX-30 µg), cefuroxime (CXM-)], fluoroquinolones [ciprofloxacin (CIP-5 µg), norfloxacin (NOR-30 µg)], nitrofurans [nitrofurantoin (NI-300 µg)], phenicols [chloramphenicol (C-30 µg)], quinolones [nalidixic acid (NA-30 µg)], sulfonamides [trimethoprim/sulphamethoxazole (TS-25 µg/25 µg)], and tetracyclines [tetracycline (T-30 µg), doxycycline (DXT-30 µg) (Mast Diagnostics, Merseyside, UK)].

### 2.5. Evaluation of Multiple Antibiotic Resistance Phenotypes (MARPs) and Multiple Antibiotic Resistance Indices (MARIs)

The multiple antibiotic-resistant phenotypes (MARPs) of the organisms were accessed for isolates that exhibited resistance against three or more antimicrobial agents following the method adapted from [41]. The multiple antibiotic resistance index (MARI) for each multiple drug-resistant (MDR) isolates was generated using the following mathematical equation adapted from [41]:

MAR index = a/b,(1)

where ‘a’ represents the number of antimicrobial agents to which the isolates exhibit resistance against and ‘b’ represents the total number of antimicrobial agents against which each isolate was tested. MARI that is equal to or greater than 0.2 shows that antimicrobial agents are indiscriminately used in that area, thus encouraging the emergence of antibiotic resistance [42].

### 2.6. Screening for Antimicrobial Resistance Genes

Following the antibiotic susceptibility testing, identified isolates that exhibited phenotypic resistance against the test antimicrobials were screened for the relevant antibiotic resistance encoding genes using the PCR technique. A total of 19 resistance genes that code for tetracycline, sulfonamide, phenicol, and aminoglycoside resistance were explored in the study using either simplex, duplex, or multiplex PCR. These genes, their sequence, cycling protocols, and anticipated amplicon sizes are presented in Supplementary Table S3. In addition, a total of 21 genes that code for *Amp*C β-lactamases and various variants of extended-spectrum β-lactamases (ESBLs) including plasmid-mediated *Amp*C and carbapenemases were also screened for using simplex and multiplex PCR as described in our previous report [43] and [44], respectively. The genes, their sequences, and expected amplicon sizes are as presented in Supplementary Table S4. The cycling conditions used for the PCR amplification of the *Amp*C β-lactamase gene is as follows; initial denaturation for 4 min at 94 °C, followed by 30 cycles of denaturation at 94 °C for 45 s, annealing for 45 s at 60 °C, extension for 45 s at 72 °C, and then final elongation for 7 min at 72 °C. For ESBL genes, the cycling conditions are as follows; initial denaturation for 10 min at 94 °C, followed by 30 cycles of denaturation at 94 °C for 40 s, annealing for 40 s at 60 °C, extension for 1 min at 72 °C, and then final elongation for 7 min at 72 °C. The annealing temperature for *bla*_OXA-48_ and *bla*_GES_ was optimal at 57 °C, and optimal at 55 °C for *bla*_KPC_, *bla*_VIM_, and *bla*_IMP_ carbapenemases. All the PCR and electrophoresis were carried out as described above.

### 2.7. Evaluation of the Patterns of Multiple Antibiotic Resistance Genotypes (MARGs)

The patterns of MARGs in the isolates harboring multiple resistance genes ≥2 were evaluated as described in our previous report [43].

### 2.8. Data Analysis

Data obtained from this study were subjected to descriptive statistical analysis using IBM Statistical Package for Social Sciences (SPSS version 21, IBM Corp., Armonk, NY, USA).

## 3. Results and Discussion

### 3.1. Antimicrobial Susceptibility Profiles of Members of Enterobacteriales

A total of 520 presumptive Enterobacterial isolates were recovered from the irrigation water and agricultural soil samples. Of these, 13 (8%) out of 160 presumptive *Salmonella* spp. were confirmed as *S.* Typhimurium, 58 (32%) out of 180 presumptive *Enterobacter* spp. were confirmed as *E. cloacae*, and 36 (20%) and 21 (12%) out of 180 presumptive *Klebsiella* spp. were confirmed as *K. pneumoniae* and *K. oxytoca*, respectively.

The antibiotic susceptibility profiles of confirmed members of Enterobacteriales isolated from irrigation water and agricultural soil to test antimicrobial agents are summarized in Table 1. According to the results, *S.* Typhimurium was susceptible to some test antimicrobial agents in frequencies that range from 7.7% for tetracycline to 100% for gentamicin, amikacin, imipenem, meropenem, ciprofloxacin, norfloxacin, and chloramphenicol. Alternatively, a high proportion of the isolates conferred resistance against ampicillin (69.2%), and tetracycline (92.3%). *E. cloacae* were susceptible to some of the test antimicrobial agents in frequencies that range from 10.3% for ampicillin and cefuroxime to 100% for amikacin and imipenem. In addition, most of the isolates were susceptible to gentamicin (98.3%), meropenem (91.4%), ciprofloxacin (86.2%), and norfloxacin (84.5%). Conversely, a high proportion of *E. cloacae* conferred resistance against amoxicillin/clavulanic acid (77.6%), ampicillin (84.5%), cefuroxime (81.0%), nitrofurantoin (81.0%), and tetracycline (60.3%). *Enterobacter* spp. are known to naturally harbor the chromosomal *Amp*C, which results in their intrinsic resistance against most antimicrobial agents. *K. pneumoniae* were susceptible to some of the test antimicrobial agents in frequencies that range from 2.8% for cefuroxime to 100% for amikacin. Most of the isolates were also susceptible to gentamicin (97.2%), imipenem (83.3%), meropenem (91.7%), ciprofloxacin (86.1%), and norfloxacin (86.1%). However, a high proportion of the isolates conferred resistance against amoxicillin/clavulanic acid (80.6%), ampicillin (88.9%), and cefuroxime (61.1%). Finally, *K. oxytoca* were susceptible to some test antimicrobial agents in frequencies that range from 4.8% for cefuroxime to 100% for gentamicin, amikacin, and imipenem. Most of the isolates were also susceptible to meropenem (90.5%), and ciprofloxacin (71.4%). Alternatively, most of the isolates conferred resistance to some test antimicrobial agents in frequencies that range from 52.4% for chloramphenicol to 61.9% for amoxicillin/clavulanic acid, ampicillin, and nitrofurantoin. This suggests that antimicrobial agents like gentamicin, amikacin, ciprofloxacin, norfloxacin, and carbapenems might still be valid for the treatment of infections caused by MDR Enterobacteria in South Africa. Precautions on the usage of these drugs should be implemented to avoid the emergence of drug resistance.

The resistance patterns of members of Enterobacteriales in this study, especially against antimicrobial agents like ampicillin, amoxicillin/clavulanic acid, tetracycline, cefuroxime, nitrofurantoin, and chloramphenicol, is similar to the findings of [18], where a high proportion of the Enterobacteria isolated from farm produce in Kentucky conferred resistance against ampicillin and tetracyclines. These antimicrobial agents belong to the beta-lactams, tetracyclines, cephalosporins, nitrofurans, and phenicols, which are thought to be the most effective classes of antimicrobial agents used for the remediation of infections induced by Enterobacteria [45], thus posing serious risks to clinical medicine. Unfortunately, the indiscriminate use of some of these antimicrobial agents in medical and veterinary settings has caused the wide distribution of broken antimicrobial agents and antibiotic resistance genes in different environmental niches including the soil, water, sludge, and sediments, consequently encouraging the rapid emergence of antimicrobial resistance (AMR) [31,46]. The global increase in the prevalence of AMR among Enterobacteria has amplified the rate of patient’s morbidity and mortality, high healthcare costs, and constant use of last-line antimicrobial agents [47].

### 3.2. The Patterns of Multiple Antibiotic Resistance Phenotypes (MARPs) and Multiple Antibiotic Resistance Indices (MARI) in the Isolates

The patterns of MAR phenotypes and the MAR indices of members of Enterobacteriales recovered from irrigation water and agricultural soil samples are presented in Table 2 and Table 3, respectively.

In irrigation water samples, *S.* Typhimurium, *E. cloacae*, *K. pneumoniae*, and *K. oxytoca* exhibited 5, 27, 16, and 10 patterns of MARPs respectively to antimicrobial agents that range from 3 to 13, many of which occurred uniquely. Although very few patterns of MARPs occurred in duplicates and triplicates as shown in Table 3. In agricultural soil samples, *S.* Typhimurium, *E. cloacae*, *K. pneumoniae*, and *K. oxytoca* exhibited 2, 16, 5, and 2 patterns of MARPS respectively to antimicrobial agents that range from 3 to 9, many of which occurred uniquely and only a few occurred in duplicate. The MAR index of all the MDR isolates recovered from irrigation water ranged from 0.2 to 0.8, and that from agricultural soil samples ranged from 0.2 to 0.6. The permissible benchmark for MARI is 0.2.

These results indicate that members of Enterobacteriales recovered in this study exhibited a high level of multidrug resistance against the test antimicrobial agents. This is probably due to antibiotic resistance selective pressure in the environment, caused by the misuse of antimicrobial agents for therapeutic and metaphylactic reasons. The results of this present study corroborate with the findings of [18], where 18.2% of the Enterobacteria isolated from farm produce displayed MDR to 10 antimicrobial agents.

MDR pathogens employ several mechanisms to resist the effects of antimicrobial agents. These pathogens may accumulate multiple genes, each coding for resistance to a single drug, or may increase the expression of genes that code for multidrug efflux pumps, extruding a wide range of antimicrobial agents. In irrigation water samples and agricultural soil samples, the highest MDR to antimicrobial agents tested was observed in *E. cloacae*. Since this pathogen is one of the most abundant Enterobacteria in the environment, its ability to acquire more resistance mechanisms is higher. In addition, *E. cloacae* are known to intrinsically overexpress *Amp*C, hence their ability to confer resistance against various classes of antimicrobial agents. This is worrisome as they are often implicated in infections like bacteremia, UTIs, intra-abdominal infections, and so on. Observing MDR in other Enterobacteria like *K. pneumoniae*, *K. oxytoca*, and *S.* Typhimurium poses a threat to the management of infections caused by these pathogens including soft tissue infections, meningitis, pneumonia, diarrhea, septicemia, and urinary tract infections [48].

Most of the patterns of MARPs were observed uniquely in all MDR members of Enterobacteriales, suggesting that these isolates must have accumulated unique and varying resistance determinants on mobile genetic elements. The predominant MARPs was “AUG-AP-CXM-NI-C-PB-CO-NA-T-DXT” and “AUG-AP-CTX-CXM-NI-C-PB-CO-NA-T-DXT”, both occurring in *E. cloacae* isolated from irrigation water samples. This suggests that there appears to be similarity in the origin and history of antibiotic exposure among the *E. cloacae* isolates.

MARI of all the MDR isolates from irrigation water and agricultural soil was ≥0.2, suggesting that the isolates are from environments where antimicrobial agents are indiscriminately used, and antibiotic resistance selective pressures are high. They thus serve as high-risk sources of multidrug-resistant pathogens and /or resistance genes to the food web.

### 3.3. Antimicrobial Resistance Genes in Phenotypically Resistant Members of Enterobacteriales

**Figure 1:**
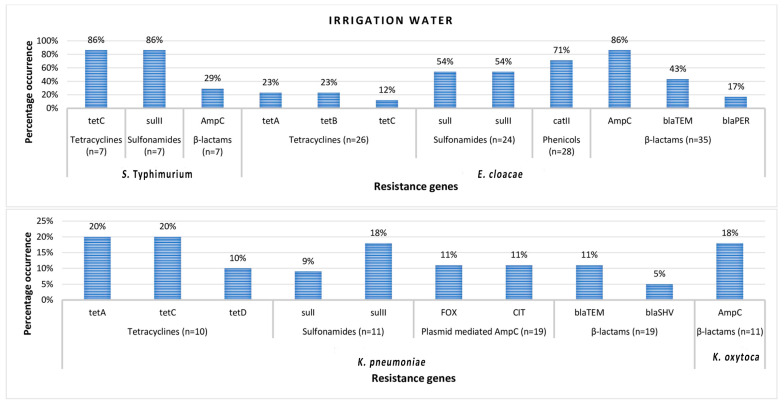
The percentage occurrence of antimicrobial resistance genes detected in confirmed members of Enterobacteriales recovered from irrigation water samples. *n* = number of phenotypically resistant isolates screened for resistance genes.

**Figure 2:**
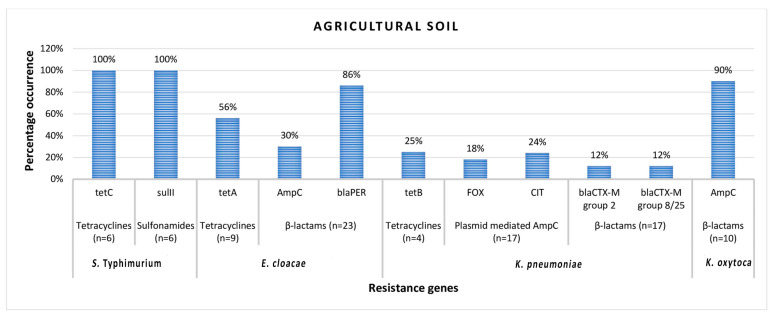
The percentage occurrence of antimicrobial resistance genes detected in confirmed members of Enterobacteriales recovered from agricultural soil samples. *n* = number of phenotypically resistant isolates screened for resistance genes.

Antibiotic resistance genes detected in *S.* Typhimurium isolated from both irrigation water and agricultural soil samples include *tet*C tetracycline resistance-encoding gene, *sul*II sulfonamide resistance-encoding gene, and *bla*_AmpC_ β-lactam resistance-encoding gene. In *E. cloacae*, antibiotic resistance genes detected include *tet*C, *tet*B, and *tet*A tetracycline resistance encoding genes, *sul*II and *sul*I sulfonamide resistance encoding genes, and *cat*II phenicol resistance-encoding gene. *bla*_AmpC_, *bla*_TEM_, and *bla*_PER_ β-lactam resistance encoding genes were also detected in *E. cloacae* isolates. In *K. pneumoniae,* the resistance genes detected include *tet*A, *tet*B, *tet*C, and *tet*D tetracycline resistance encoding genes, *sul*I and *sul*II sulfonamide resistance encoding genes, FOX-type and CIT-type plasmid-mediated *AmpC* β-lactam resistance encoding genes, *bla*_TEM_ and *bla*_SHV_ β-lactam resistance encoding genes, and finally *bla*_CTX-M_ group 2 and *bla*_CTX-M_ group 8/25 β-lactam resistance encoding genes. In *K. oxytoca*, only the *bla*_AmpC_ β-lactam resistance-encoding gene was detected. The frequency of occurrence of antibiotic resistance genes (ARGs) detected in members of Enterobacteriales recovered from irrigation water and agricultural soil samples are shown in Figure 1 and Figure 2, respectively.

In irrigation water samples, the frequency of detection of ARGs in the *S.* Typhimurium isolates followed the order; 86% (*tet*C), 86% (*sul*II), and 29% (*bla*_AmpC_); while for the *E. cloacae,* the order is as follows 23% (*tet*A), 23% (*tet*B), 12% (*tet*C), 54% (*sul*I), 54% (*sul*II), 71% (*cat*II), 86% (*bla*_AmpC_), 43% (*bla*_TEM_), and 17% (*bla*_PER_). For *Klebsiella pneumonia*, the order is 20% (*tet*A), 20% (*tet*C), 10% (*tet*D), 9% (*sul*I), 18% (*sul*II), 11% (FOX-) and 11% (CIT-type plasmid-mediated *Amp*C), 11% (*bla*_TEM_) and 5% (*bla*_SH_v); and finally, 18% (*bla*_AmpC_) occurred in *K. oxytoca*.

In agricultural soil samples, all the *S.* Typhimurium isolates habored *tet*C and *sul*II. The frequency of detection of ARGs was 56% (*tet*A), 30% (*bla*_AmpC_), and 86% (*bla*_PER_) in *E. cloacae*; 25% (*tet*B), 18% (FOX- type plasmid-mediated *AmpC*), 24% (CIT- type plasmid-mediated *AmpC*), 12% (*bla*_CTX-M_ group 2), and 12% (*bla*_CTX-M_ group 8/25) in *K. pneumoniae*; and finally, 90% (*bla*_AmpC_) in *K. oxytoca*.

The high prevalence of ARGs like the tetracycline-encoding genes, sulfonamide-encoding genes, and the ESBLs shows that the tetracycline, sulfonamide, and beta-lactam classes of antimicrobial agents are more frequently used in the Eastern Cape Province of South Africa, where this study was carried out. Studies have shown that these classes of antimicrobial agents are usually used as first-line antimicrobial agents in human and veterinary medicine, even as growth promoters in animal husbandry [49]. Usually, most of these antimicrobial agents are poorly absorbed, while the rest are discharged in the environment where they continue to encourage the emergence of AMR. Enterobacteria plays an important role in the current dissemination of ARGs from environmental bacteria to human pathogens and vice versa [50,51].

Among the ESBLs, *bla*_PER_ in *E. cloacae* recovered from agricultural soil samples had the highest prevalence. This was followed by *bla*_TEM_ in *S.* Typhimurium recovered from irrigation water samples. The prevalence of *bla*_AmpC_ was also high in the study, as all the isolates harbored it apart from *K. pneumoniae*. The dominance of the different types of ESBLs and *Amp*C enzymes varies with geographical locations and countries as each country has a unique way of dispensing different classes of antimicrobial agents. For instance, CTX-M is dominant in China and also reported to be the most common ESBL worldwide [52]. The TEM-, SHV-, and CTX-M- types are more dominant in Europe and the US [53]. The SHV-type is dominant in Japan [54]. Molecular characterization as consistently reported in other studies revealed that the TEM-, followed by the CTX-M-type, and then SHV are dominant in other countries [55,56].

All these resistance genes including the ESBLs harbored by members of Enterobacteriales in this study are correlated to the high level of phenotypic resistance of the isolates against antimicrobial agents like tetracyclines, chloramphenicol, and beta-lactams like ampicillin, amoxicillin/clavulanic acid, and cefuroxime. In the 1980s, the SHV- and TEM-type ESBLs were believed to be the cause of resistance to third-generation cephalosporins like cefuroxime and cefotaxime in Enterobacteria [57]. In the 2000s, the tides turned as the CTX-M-type ESBL became dominant over the TEM- and SHV-type enzymes [52]. The carbapenemases including *bla*_VIM_, *bla*_IMP_, and *bla*_KPC_ were not detected in any of the members of Enterobacteriales in this study. This probably explains why the low level of phenotypic resistance against imipenem and meropenem was observed. Studies have shown that carbapenems are the most effective group of antimicrobial agents against Gram-positive and Gram-negative bacteria, presenting a broad spectrum of antibacterial activity. This is attributed to their unique molecular structure which contains both carbapenems and beta-lactam ring assisting in their exceptional stability against most beta-lactamases (enzymes that inactivate beta-lactams) including *Amp*C and ESBLs [58]. This explains why they are considered as antibiotics of last resort, hence must be prudently used.

In general, the results showed that ARGs detected in the present study suggested their implications in the resistance characteristics. It also suggests that the resistance observed in the isolates were probably acquired via horizontal gene transfer since acquired resistance mechanisms often result in a predictable increase in phenotypic resistance. This is detrimental to public health, as these ARGs will continue to be shared among related and unrelated bacteria within various niches of the agro-ecosystem and the environment, and even extend to clinically relevant pathogens, making them potential pan-resistant bacteria.

### 3.4. The Patterns of Multiple Antibiotic Resistance Genotypes (MARGs) in the Isolates

The patterns of multiple antibiotic resistance genotypes (MARGs) in this study are shown in Table 4 and Table 5, respectively.

In irrigation water, *S.* Typhimurium exhibited 3 patterns of MARGs while *E. cloacae* and *K. pneumoniae* exhibited 16 and 5 patterns of MARGs respectively, made up of both β-lactamases and non β-lactamases most of which were uniquely observed. “*tet*C*- sulII*” in *S.* Typhimurium was the most frequently observed MARGs in irrigation water samples, which occurred 3 times. *K. oxytoca* did not exhibit MARGs. The highest MARGs detected was in *E. cloacae*, which included 6 ARGs (*tet*A*- tet*B*- sulI- sulII- catII- bla*_TEM_) made up of 1 ESBL and 5 non-ESBL.

In agricultural soil samples, *S.* Typhimurium, *E. cloacae*, and *K. pneumoniae* exhibited 1, 2, and 2 patterns of MARGs respectively, some of which were made up of both beta-lactamases and non-beta lactamases and were uniquely observed. The highest MARGs was observed in *K. pneumoniae*, made up of 3 ARGs of which they were all ESBLs (*bla*_FOX_*- bla*_CIT_*- bla*_CTX-M_ group 2). *tet*C*- sulII* in *S.* Typhimurium was the most frequently observed MARGs in agricultural soil samples which occurred 6 times. MARGs was not observed in *K. oxytoca.*

In this study, members of Enterobacteriales harbored several different ARGs. This co-occurrence of ARGs enabled the isolate to confer resistance against multiple antimicrobial agents. This situation is probably due to the sequential exposure of the isolates to multiple antimicrobial agents in high-risk environments [59]. The more the isolates are exposed to sublethal concentrations of antimicrobial agents in the environment, the more they acquire a variety of ARGs [59]. Most of the MARGs were observed in *E. cloacae* recovered from irrigation water and agricultural soil. *E. cloacae* are widely distributed in the environment hence their high rate of exposure to several antimicrobial agents and the subsequent acquisition of multiple ARGs. *tet*C*- sulII* was the most prevalent MARGs. This suggests that the environment is probably saturated with sublethal levels of tetracyclines and sulfonamides. Fewer MARGs were observed in isolates from agricultural soil compared to irrigation water samples, and this could be because the irrigation water sources were more prone to different sources of contamination. The co-occurrence of ESBLs and non-ESBLs in the isolates demonstrate the possible exchange of ARGs between the bacteria. This is detrimental to the agro-ecosystem as it could alter the microbiome of the milieu [60].

## 4. Conclusions

This study demonstrates that antibiotic-resistant Enterobacteria including *S.* Typhimurium, *E. cloacae*, *K. pneumonia*, and *K. oxytoca* with outbreak potentials occur in irrigation water and agricultural soil in the Eastern Cape Province of South Africa. Based on the cultural and molecular methods adapted, the study also demonstrates that the pathogens exhibited multidrug resistance against the test antimicrobial agents and harbored multi-genetic repertoires including the ESBLs, which aided in their resistance to test antimicrobial agents. These results confirm that irrigation water and agricultural soil, supposedly the two most important transmission routes of fresh produce associated pathogens, harbor and potentially convey antibiotic-resistant Enterobacteria to fresh produce destined for human consumption. Implementation of good agricultural practices such as erecting of fences around farms and irrigation water sources, use of properly composted manure for soil amendment, use of appropriate irrigation methods, and routine microbial testing of irrigation water and organic fertilizers by the local community will minimize the public health risks posed by these pathogens.

## Figures and Tables

**Table 1:** Antibiotic susceptibility profiles of target members of Enterobacteriales.

Antibiotics	Potency (µg)	*S.* Typhimurium *n* = 13 (Frequency/Percent)			*E. cloacae* *n* = 58 (Frequency/Percent)			*K. pneumoniae* *n* = 36 (Frequency/Percent)			*K. oxytoca* *n* = 21 (Frequency/Percent)		
		R	I	S	R	I	S	R	I	S	R	I	S
Gentamicin	10	0/0	0/0	13/100	1/1.7	0/0	57/98.3	1/2.8	0/0	35/97.2	0/0	0/0	21/100
Amikacin	30	0/0	0/0	13/100	0/0	0/0	58/100	0/0	0/0	36/100	0/0	0/0	21/100
Amoxicillin/ Clavulanic acid	20/10	5/38.5	2/15.4	6/46.2	45/77.6	2/3.4	11/19.0	29/80.6	3/8.3	4/11.1	13/61.9	0/0	8/38.1
Ampicillin	10	9/69.2	0/0	4/30.8	49/84.5	3/5.2	6/10.3	32/88.9	0/0	4/11.1	13/61.9	0/0	8/38.1
Imipenem	10	0/0	0/0	13/100	0/0	0/0	58/100	2/5.6	4/11.1	30/83.3	0/0	0/0	21/100
Meropenem	10	0/0	0/0	13/100	1/1.7	4/6.9	53/91.4	0/0	3/8.3	33/91.7	0/0	2/9.5	19/90.5
Cefotaxime	30	2/15.4	0/0	11/84.6	30/51.7	6/10.3	22/37.9	16/44.4	3/8.3	17/47.2	10/47.6	0/0	11/52.4
Cefuroxime	30	5/38.5	8/61.5	0/0	47/81.0	5/8.6	6/10.3	22/61.1	13/36.1	1/2.8	12/57.1	8/38.1	1/4.8
Ciprofloxacin	5	0/0	0/0	13/100	1/1.7	7/12.1	50/86.2	4/11.1	1/2.8	31/86.1	3/14.3	3/14.3	15/71.4
Norfloxacin	30	0/0	0/0	13/100	2/3.4	7/12.1	49/84.5	4/11.1	1/2.8	31/86.1	5/23.8	2/9.5	14/66.7
Nitrofurantoin	300	1/7.7	1/7.7	11/84.6	47/81.0	2/3.4	9/15.5	18/50.0	2/5.6	16/44.4	13/61.9	0/0	8/38.1
Chloramphenicol	30	0/0	0/0	13/100	32/55.2	10/17.2	16/27.6	15/41.7	2/5.6	19/52.8	11/52.4	2/9.5	8/38.1
Nalidixic acid	30	2/15.4	0/0	11/84.6	28/48.3	4/6.9	26/44.8	14/38.9	2/5.6	20/55.6	8/38.1	0/0	13/61.9
Trimethoprim/ Sulfamethoxazole	1.25/23.75	3/23.1	0/0	10/76.9	29/50.0	1/5.2	26/44.8	10/27.8	1/2.8	25/69.4	8/38.1	0/0	13/61.9
Tetracycline	30	12/92.3	0/0	1/7.7	35/60.3	5/8.6	18/31.0	13/36.1	2/5.6	21/58.3	8/38.1	1/4.8	12/57.1
Doxycycline	30	2/15.4	7/53.8	4/30.8	32/55.2	10/17.2	16/27.6	7/19.4	12/33.3	17/47.2	2/9.5	6/28.6	13/61.9

R: Resistant, I: Intermediate, S: Susceptible; *n*: number of isolates tested.

**Table 2:** Patterns of MAR phenotypes and MAR index of members of Enterobacteriales isolated from irrigation water samples.

SN	MAR Phenotypes	No. of Antibiotics	No. of Isolates	MARI
	*S.* Typhimurium			
1	AUG-AP-CTX-CXM-NI-T	6	1	0.4
2	CTX-CXM-T-DXT	4	1	0.3
3	AP-CXM-T	3	1	0.2
4	AUG-AP-NA-T-DXT	5	1	0.3
5	AUG-AP-CXM-T	4	1	0.3
	*E. cloacae*			
1	AUG-AP-CXM-NI-C-NA-T-DXT	8	3	0.5
2	AUG-AP-CXM-NI-NA-T-DXT	7	1	0.4
3	AUG-AP-CTX-CXM-CIP-NI-NA-T-DXT	9	1	0.6
4	AUG-CXM-NI-NA-T-DXT	6	1	0.4
5	AUG-AP-CXM-NI-T-DXT	6	1	0.4
6	AUG-AP-CTX-CXM-NI-C-T-DXT	8	1	0.5
7	AUG-AP-CTX-CXM-NI-C-T-DXT	8	1	0.5
8	AUG-CXM-NI-C	4	1	0.3
9	AUG-AP-CXM-NI-C	5	2	0.3
10	AUG-AP-CTX-CXM-NI-C	6	1	0.4
11	AP-CXM-NI-C-T-DXT	6	1	0.4
12	AUG-AP-CXM-NOR-NI-C-NA-T-DXT	9	1	0.6
13	AP-CXM-NI-C-T-DXT	6	1	0.4
14	AP-NI-C-T-DXT	5	1	0.3
15	AP-CXM-NI-C-T-DXT	6	1	0.4
16	AP-CTX-CXM-NI-C	5	1	0.3
17	AP-CTX-CXM-NI-C-NA-T-DXT	8	2	0.5
18	AUG-AP-CTX-CXM-NI-C-NA-T-DXT	9	1	0.6
19	AUG-AP-CTX-CXM-NI-C-NA-T	8	1	0.5
20	AUG-AP-CTX-CXM-NI-NA-TS-T-DXT	9	1	0.6
21	AUG-AP-CTX-CXM-NI-NA-T-DXT	8	1	0.5
22	AUG-AP-CTX-CXM-NI-C-T-DXT	8	1	0.5
23	AUG-AP-CTX-CXM-NOR-NI-C-T-DXT	9	1	0.6
24	AUG-AP-CTX-CXM-NI-C-NA-T-DXT	9	3	0.6
25	AUG-AP-CTX-CXM-NI-C-NA	7	2	0.4
26	GM-AUG-AP-MEM-CTX-CXM-NI-C-NA	9	1	0.6
27	AUG-NI-C-T-DXT	5	1	0.3
	*K. pneumoniae*			
1	AUG-AP-CTX-CXM-NI-C-NA	7	1	0.4
2	AUG-AP-IMI-CTX-CXM-NI C-NA	8	1	0.5
3	AUG-AP-CTX-CXM-NI-C	6	1	0.4
4	AUG-AP-CTX-CXM-CIP-NOR-NI-C-NA-TS-T	11	2	0.7
5	AUG-AP-IMI-CXM-NI-C-NA-TS-T	9	1	0.6
6	AUG-AP-CTX-CXM-NI-C-NA-TS-T	9	1	0.6
7	AP-CTX-CXM-NI-C-NA-TS-T	8	1	0.5
8	GM-AUG-AP-MEM-CTX-CXM-NI-C-NA-TS-DXT	11	1	0.7
9	AUG-AP-MEM-CTX-CXM-NOR-NI-NA-TS	9	1	0.6
10	AUG-AP-MEM-CTX-CXM-CIP-NOR-C-NA-TS-T-DXT	12	1	0.8
11	AUG-AP-CXM-NI-C-NA-TS-T-DXT	9	1	0.6
12	AUG-AP-CTX-CXM-NI-C-NA-TS-T-DXT	10	1	0.6
13	AUG-AP-NI	3	2	0.2
14	AUG-AP-CTX-CXM-NI-T	6	1	0.4
15	AUG-AP-CTX-CXM-NA	5	1	0.3
16	AUG-AP-CXM	3	1	0.2
	*K. oxytoca*			
1	AUG-AP-CTX-CXM-CIP-NI -C-NA-TS	9	1	0.6
2	AUG-AP-CTX-CXM-NI-C-NA-TS-T-DXT	10	1	0.6
3	AUG-AP-CTX-CXM-NOR-NI-C-T	8	1	0.5
4	AUG-AP-CTX-CXM-NOR-NI-C-NA-TS-T	10	1	0.6
5	AUG-AP-CTX-CXM-CIP-NOR-NI-C-NA-TS-T	11	1	0.7
6	AUG-AP-CTX-CXM-CIP-NOR-NI-C-NA-TS-T	11	1	0.7
7	AUG-AP-CTX-CXM-NI-C-T	7	1	0.4
8	AUG-AP-CXM-NI-T	5	1	0.3
9	AUG-AP-CTX-CXM-NOR-NI-C-NA-TS-T	10	1	0.6
10	AUG-AP-CTX-CXM-NI-C-NA-TS	8	1	0.5

SN—serial number, GM—gentamycin, AK—amikacin, AUG—amoxicillin/clavulanic acid, AP—ampicillin, IMI—imipenem, MEM—meropenem, CTX—cefotaxime, CXM—cefuroxime, CIP—ciprofloxacin, NOR—norfloxacin, NI—nitrofurantoin, C—chloramphenicol, NA—nalidixic acid, TS—trimethoprim/sulphamethoxazole, T—tetracycline, DXT—doxycycline.

**Table 3:** Patterns of MAR phenotypes and MAR index of members of Enterobacteriales isolated from agricultural soil samples.

SN	MAR Phenotypes	No. of Antibiotics	No. of Isolates	MARI
	*S.* Typhimurium			
1	AUG-AP-T	3	2	0.2
2	AP-NA-T	3	1	0.2
	*E. cloacae*			
1	AUG-NA-T-DXT	4	1	0.3
2	AUG-AP-T	3	1	0.2
3	AUG-AP-CTX-CXM-NI-C-NA-T-DXT	9	1	0.6
4	AUG-AP-NI	3	1	0.2
5	AUG-AP-CXM	3	2	0.2
6	AUG-AP-CTX-CXM-NI-C	6	1	0.4
7	AUG-AP-CTX-CXM-NI	5	1	0.3
8	AP-CXM-NI-T	4	1	0.3
9	PB-T-DXT	3	1	0.2
10	AUG-AP-CXM-C-T-DXT	6	1	0.4
11	AUG-AP-CTX	3	1	0.2
12	AUG-AP-CTX-CXM-NI-NA-T-DXT	8	2	0.5
13	AUG-AP-CTX-CXM-NI-NA	6	2	0.4
14	AUG-AP-CTX-CXM-NI-NA-TS-T-DXT	9	1	0.6
15	AUG-AP-CTX-CXM-NI-NA-TS	7	1	0.4
16	CXM-NI-C	3	1	0.2
	*K. pneumoniae*			
1	AUG-AP-T-DXT	4	1	0.3
2	AUG-AP-CTX-CXM-NI-C-T-DXT	8	2	0.5
3	AUG-AP-CTX-CXM-NI-C-NA-T	8	1	0.5
4	AUG-AP-CIP	3	1	0.2
5	AUG-AP-CXM	3	2	0.2
	*K. oxytoca*			
1	AUG-AP-CTX-CXM-NI-NA-TS-DXT	8	1	0.5
2	AUG-AP-CXM	3	1	0.2

SN—serial number, GM—gentamycin, AK—amikacin, AUG—amoxicillin/clavulanic acid, AP—ampicillin, IMI—imipenem, MEM—meropenem, CTX—cefotaxime, CXM—cefuroxime, CIP—ciprofloxacin, NOR—norfloxacin, NI—nitrofurantoin, C—chloramphenicol, NA—nalidixic acid, TS—trimethoprim/sulphamethoxazole, T—tetracycline, DXT—doxycycline.

**Table 4:** Patterns of MAR genotypes of Enterobacteria isolated from irrigation water samples.

SN	MAR Genotypes	No. of Resistance Genes other than β-lactamases	No. of β-lactamases	No. of Observed Pattern
	*S.* Typhimurium			
1	*tet*C*- sulII*	2	0	3
2	*tet*C*- bla*_AmpC_	1	1	1
3	*tet*C *-sulII- bla*_AmpC_	2	1	1
	*E. cloacae*			
1	*tet*A*- tet*B*- sulI- sulII- catII- bla*_TEM_	5	1	1
2	*tet*A*- tet*B*- sulI- sulII*	4	0	1
3	*tet*A*- tet*B*- sulI- sulII- bla*_TEM_	4	1	2
4	*tet*A*- sulI- sulII- bla*_TEM_	3	1	1
5	*tet*A*- tet*B*- bla*_TEM_	2	1	1
6	*tet*C*- sulI- sulII- catII- bla*_PER_	4	1	1
7	*tet*C*- catII- bla*_TEM_*- bla*_PER_	2	2	1
8	*bla*_TEM_ *- bla*_PER_	0	2	1
9	*catII- bla*_TEM_ *- bla*_PER_	1	2	1
10	*tet*C*- sulI- sulII- catII- bla*_TEM_	4	1	1
11	*sulI- sulII- catII- bla*_TEM_	3	1	1
12	*tet*B*-sulI-sulII-catII- bla*_AmpC_*- bla*_TEM_	4	2	1
13	*sulI- catII- bla*_TEM_	2	1	1
14	*sulI- sulII- catII- bla*_TEM_	3	1	1
15	*catII- bla*_TEM_ *- bla*_PER_	1	2	1
16	*sulI- sulII- catII*	3	0	2
	*K. pneumoniae*			
1	*bla*_FOX_ *- bla*_CIT_	0	2	2
2	*tet*D*- sulI- bla*_TEM_	2	1	1
3	*sulII- bla*_TEM_	1	1	1
4	*tet*A*- bla*_SHV_	1	1	1
5	*tet*A*- tet*C	2	0	1

**Table 5:** Patterns of MAR genotypes of Enterobacteria isolated from agricultural samples.

SN	MAR Genotypes	No. of Resistance Genes other than β-lactamases	No. of β-lactamases	No. Observed
	*S.* Typhimurium			
1	*tet*C*- sulII*	2	0	6
	*E. cloacae*			
1	*bla*_AmpC_ *- bla*_PER_	0	2	1
2	*tet*A*- bla*_AmpC_	1	1	1
	*K. pneumoniae*			
1	*bla*_FOX_ *- bla*_CIT_ *- bla*_CTX-M_^a^	0	3	1
2	*bla*_FOX_ *- bla*_CIT_	0	2	2

Key: a = group2.

## Supplementary Materials

The following are available online at https://www.mdpi.com/2076-2607/8/8/1206/s1, Table S1: Description of sampling points, Table S2: Primer sequence and PCR cycling conditions used for the molecular detection of members of Enterobacteriales, Table S3: The primer sequence and expected amplicon size used for the screening of resistance genes in members of Enterobacteriales, Table S4: The primer sequence and expected amplicon size used for the screening of *Amp*C β-lactamase and ESBLs in members of Enterobacteriales [44].
