# Supplementary material for: Antibiogram Signatures of Some Enterobacteria Recovered from Irrigation Water and Agricultural Soil in two District Municipalities of South Africa
Source: Microorganisms. 2020 Aug 7;8(8):1206. doi: 10.3390/microorganisms8081206 (PMC7463487; doi:10.3390/microorganisms8081206)
Supplement: Supplementary file 1 [file microorganisms-08-01206-s001.pdf]

Table S1: Description of sampling points.

District Municipality	Sample sites	Samples collected	Description of samples	Anthropogenic activities
Amathole	S1	Irrigation water	Irrigation water is sourced from a river. Soil samples were not collected due to the inaccessibility of agricultural farms	Animal intrusion, dumping of refuse, recreational activities, swimming, and domestic activities in the source river.
	S2	Irrigation water and soil	Irrigation water is sourced from a dam. Type of irrigation used is a sprinkler irrigation system. Irrigation is done once in 3 days between 8 am and 3 pm on each day. Soil type is loam and crops grown on it include cabbage, broccoli, butternut, spinach and lettuce.	Animal intrusion in the dam. Soil amendment is with inorganic fertilizer (LAN).
	S3	Irrigation water and soil	Irrigation water is sourced from an artificial pond which receives water from both rainfall and downstream river. Type of irrigation used is a sprinkler irrigation system. Irrigation is done once a day between 8 am and 12 pm, and none on rainy days. Soil type is clay-loam and crops grown on it include cabbage, broccoli, lettuce and onions.	Animal intrusion in the pond and source river. Soil amendment is with livestock waste.
	S4	Irrigation water	Irrigation water is sourced from a dam. Soil samples were not collected due to the inaccessibility of agricultural farms	Animal intrusion and domestic use of water.
	S5	Irrigation water and soil	Irrigation water is sourced from an artificial pond, which primarily receives water from the effluents of a poultry and piggery farm. Type of irrigation used is the sprinkler irrigation system. Irrigation is done once every day between 8 am and 5 pm except on rainy days. Soil type is loam and crops grown on it include spinach and cabbage.	Discharge of swine and poultry effluents into the pond. Soil amendment is with poultry and swine faeces.
	S6	Irrigation water	Irrigation water is sourced from a river. Soil samples were not collected due to the inaccessibility of agricultural farms	Animal intrusion, recreational activities, swimming, and domestic activities in the source river.
	S7	Irrigation water and soil	Irrigation water is sourced from a river. Type of irrigation used is a sprinkler irrigation system. Irrigation is done twice a week between 8 am and 4 pm on each day. Soil type is clay and crops grown on it include pumpkin.	Animal intrusion, washing of cloths, bathing and other domestic activities in the source river. Soil amendment is with organic compost of livestock origin.

	S8	Irrigation water and soil	Irrigation water is sourced from a river. Type of irrigation used is a sprinkler irrigation system. Irrigation is done twice a day between 8 am and 12 pm and between 2 pm and 5 pm and none on days with heavy rainfall. Soil type is clay-loam and crops grown on it include cabbage, lettuce and spinach.	Animal intrusion in both river and close to farm sites. Soil amendment is with cow dung.
	S9	Irrigation water	Irrigation water is sourced from a dam. Type of irrigation used is a sprinkler irrigation system. Irrigation is done once a day and none on days with heavy rainfall.	Animal intrusion
		Soil	Soil type is loamy. Crops grown on it include lettuce and spinach.	Soil amendment is with composted animal waste.
	S10	Irrigation water	Irrigation water is sourced from a river. Soil samples were not collected due to the inaccessibility of agricultural farms	Animal intrusion, washing of cloths, and fishing.
	S11	Irrigation water and soil	Irrigation water is sourced from a river and manually irrigated on a backyard farm using watering cans. Soil type is loam and crops grown on it include spinach.	Animal intrusion, swimming, and washing of clothes in the source river. No soil amendment
	S12	Irrigation water and soil	Irrigation water is sourced from treated municipal water. Type of irrigation used is a sprinkler irrigation system. Irrigation is done once a day from 8 am to 1 pm. Soil type is clay-loam and crops grown on it include cabbage, lettuce, onions and spinach.	No visible anthropogenic activity in the water source. Soil amendment is done using inorganic fertilizer (LAN).
	S13	Irrigation water and soil	Irrigation water is sourced from a river. Type of irrigation used is the sprinkler irrigation system. Irrigation is done once a day from 8 am to 12 pm. Soil type is clay-loam and crops grown on it include cabbage, broccoli, lettuce and spinach.	Animal intrusion in the river. Soil amendment is with inorganic fertilizer (LAN).
	S14	Irrigation water and soil	Irrigation water is sourced from a dam. Type of irrigation used is a sprinkler irrigation system. Irrigation is done once a day. Soil type is clay and crops grown on it include cabbage, lettuce and spinach.	No observed anthropogenic activity in the dam. Soil amendment is with inorganic fertilizer (LAN).
Chris Hanni	S15	Irrigation water	Irrigation water is sourced from a river. Soil samples were not collected due to the inaccessibility of agricultural farms	Animal intrusion
	S16	Irrigation water and soil	Irrigation water is sourced from a river. Type of irrigation used is the sprinkler irrigation system. Irrigation is done	Animal intrusion. Soil amendment is with organic compost of animal origin

		once a day. Soil type is loam and crops grown on it include lettuce and spinach.	
S17	Irrigation water and soil	Irrigation water is sourced from borehole groundwater. Types of irrigation used include sprinkler and drip irrigation system. Irrigation is done twice a day. Soil type is clay-loam and crops grown on it include cabbage, lettuce, broccoli and spinach.	No anthropogenic activity in the water source. Soil amendment is with inorganic fertilizer (LAN).
S18	Irrigation water and soil	Irrigation water is sourced from a river. Type of irrigation used is the center pivot irrigation system. Irrigation is done once a day. Soil type is loam and crops grown on it is fodder	Animal intrusion, receiving shed of WWTP effluent. Soil amendment is with inorganic fertilizer (LAN)
S19	Irrigation water	Irrigation water is sourced from a river. Soil samples were not collected due to the inaccessibility of agricultural farms	Animal intrusion, dumping of refuse.

Note: For confidentiality sake, sampling sites are coded with S1-S19. All the information recorded in this table were gotten from the onsite survey as well as from farm owners and workers. Coordinates for each sampling site were retrieved using the "etrex-LEGENDH" GPS equipment. WWTP: Wastewater treatment plant. LAN: limestone ammonium nitrogen.

Table S2: Primer sequence and PCR cycling conditions used for the molecular detection of members of Enterobacteriales

Target organisms	Target gene	Primer sequence (5′-3′)	PCR cycling conditions	Amplicon size (bp)	Reference
Salmonella genus	*invA*	F: TATCGCCACGTTCGGGCAA<br>R: TCGCACCGTCAAAGGAACC	95 °C, 2min; 35[95 °C, 20 s; 57 °C, 15 sec; 72 °C, 2 min] 72 °C, 7 min	275	[2]
	ompC	F: ATCGCTGACTTATGCAATCG<br>R: CGGGTTGCGTTATAGGTCTG	95 °C, 2min; 35[95 °C, 20 s; 57 °C, 15 sec; 72 °C, 2 min] 72 °C, 7 min	204	[3]
Salmonella enterica serovar Typhimurium	*typh*	F: TTGTTCACTTTTTACCCCTGAA<br>R: CCCTGACAGCCGTTAGATATT	95 °C, 2min; 30[95 °C, 1 min; 57 °C, 1 min; 72 °C, 2 min] 72 °C, 5 min	401	[4]
Enterobacter cloacae	*hsp60*	F: GTAGAAGAA GGCGTGGTTGC<br>R: ATGCATTCGGTGGTGATCATCAG	95 °C, 5 min; 30[94°C, 30 s; 57 °C, 30 s; 72 °C, 60 s] 72 °C, 5 min	341	[5]
Klebsiella genus	*gyrA*	F: CGCGTACTATACGCCATGAACGTA<br>R: ACCGTTGATCACTTCGGTCAGG	94 °C, 5 min; 35[94 °C, 30 sec; 55 °C, 45 sec, 72 °C, 45 sec] 72 °C, 10 min	441	[6]
Klebsiella pneumoniae	*16S-23S ITS*	F: ATTTGAAGAGGTTGCAAACGAT<br>R: TTCACTCTGAAGTTTTCTTGTGTTC	94 °C, 5 min; 30[94 °C, 30 sec; 55 °C, 30 sec; 72 °C, 40 sec] 72 °C, 10 min	130	[7]
Klebsiella oxytoca	*pehX*	F: GATACGGAGTATGCCTTTACGGTG<br>R: TAGCCTTTATCAAGCGGATACTGG	94 °C, 5 min; 30[94 °C, 30 sec; 55 °C, 30 sec; 72 °C, 40 sec] 72 °C, 10 min	343	[8]

Table S3: The primer sequence and expected amplicon size used for the screening of resistance genes in members of Enterobacteriales

Antibiotic class	Genes	PCR primer sequence (5'–3')	PCR cycling condition	Amplicon size (bp)	Reference
Tetracyclines	*tet*A	F: GCTACATCCTGCTTGCCTTC<br>R: CATAGATCGCCGTGAAGAGG	94 °C,5m; 35[94 °C,1m; 55 °C,1m; 72 °C,1.5m]; 72 °C,5m	210	[9]
	*tet*B	F: TTGGTTAGGGGCAAGTTTTG<br>R: GTAATGGGCCAATAACACCG	94 °C,5m; 35[94 °C,1m; 55 °C,1m; 72 °C,1.5m]; 72 °C,5m	659	[10]
	*tet*C	F: CTTGAGAGCCTTCAACCCAG<br>R: ATGGTCGTCATCTACCTGCC	94 °C,5m; 35[94 °C,1m; 55 °C,1m; 72 °C,1.5m]; 72 °C,5m	418	[10]
	*tet*D	F: AAACCATTACGGCATTCTGC<br>R: GACCGGATACACCATCCATC	94 °C,5m; 35[94 °C,1m; 55 °C,1m; 72 °C,1.5m]; 72 °C,5m	787	[10]
	*tet*E	F: AAACCACATCCTCCATACGC<br>R: AAATAGGCCACAACCGTCAG	94 °C,5m; 35[94 °C,1m; 55 °C,1m; 72 °C,1.5m]; 72 °C,5m	278	[11]
	*tet*G	F: GCTCGGTGGTATCTCTGCTC<br>R: AGCAACAGAATCGGGAACAC	94 °C,5m; 35[94 °C,1m; 55 °C,1m; 72 °C,1.5m]; 72 °C,5m	468	[12]
	*tet*K	F: TCGATAGGAACAGCAGTA<br>R: CAGCAGATCCTACTCCTT	94 °C,5m; 35[94 °C,1m; 55 °C,1m; 72 °C,1.5m]; 72 °C,5m	169	[13]
	*tet*L	F: TCGTTAGCGTGCTGTCATTC<br>R: GTATCCCACCAATGTAGCCG	94 °C,5m; 35[94 °C,1m; 55 °C,1m; 72 °C,1.5m]; 72 °C,5m	267	[14]
	*tet*M	F: GTGGACAAAGGTACAACGAG<br>R: CGGTAAAGTTCGTCACACAC	94 °C,5m; 35[94 °C,1m; 55 °C,1m; 72 °C,1.5m]; 72 °C,5m	406	[13]
Sulfonamides	*sulI*	F: CGGCGTGGGCTACCTGAACG<br>R: GCCGATCGCGTGAAGTTCCG	94 °C,5m; 30[94 °C,30s; 65 °C,30s; 72 °C,2m]; 72 °C,10m	433	[15]

	sulII	F: GCGCTCAAGGCAGATGGCATT<br>R: GCGTTTGATACCGGCACCCGT	94 °C,5m; 30[94 °C,30s; 65 °C,30s; 72 °C,2m]; 72 °C,10m	293	[15]
Phenicols	*cmlA1*	F: CACCAATCATGACCAAG<br>R: GGCATCACTCGGCATGGACATG	94 °C,5m; 30[94 °C,30s; 50 °C,30s; 72 °C,1.5m]; 72 °C,5m	115	[16]
	catI	F: AGTTGCTCAATGTACCTATAACC<br>R: TTGTAATTCATTAAGCATTCTGCC	94 °C,5m; 30[94 °C,30s; 50 °C,30s; 72 °C,1.5m]; 72 °C,5m	320	[17]
	catII	F: ACACTTTGCCCTTTATCGTC<br>R: TGAAAGCCATCACATACTGC	94 °C,5m; 30[94 °C,30s; 50 °C,30s; 72 °C,1.5m]; 72 °C,5m	543	[17]
Aminoglycosides	*strA*	F CTTGGTGATAACGGCAATTC<br>R: CCAATCGCAGATAGAAGGC	94 °C,4m; 30[94 °C,45s; 50 °C,45s; 72 °C,45s]; 72 °C,5m	348	[1]
	aadA	F: GTGGATGGCGGCCTGAAGCC<br>R: AATGCCCAGTCGGCAGCG	94 °C,4m; 30[94 °C,45s; 50 °C,45s; 72 °C,45s]; 72 °C,5m	525	[1]
	aac(3)-IIa (aacC2)[a]	F: CGGAAGGCAATAACGGAG<br>R: TCGAACAGGTAGCACTGAG	94 °C,5m; 30[94 °C,30s; 50 °C,30s; 72 °C,1.5m]; 72 °C,5m	428	[17]
	aph(3)-Ia (aphA1)[a]	F: ATGGGCTCGCGATAATGTC<br>R: CTCACCGAGGCAGTTCCAT	94 °C,5m; 30[94 °C,30s; 50 °C,30s; 72 °C,1.5m]; 72 °C,5m	600	[17]
	aph(3)-IIa (aphA2)[a]	F: GAACAAGATGGATTGCACGC<br>R: GCTCTTCAGCAATATCACGG	94 °C,5m; 30[94 °C,30s; 50 °C,30s; 72 °C,1.5m]; 72 °C,5m	510	[17]

Table S4: The primer sequence and expected amplicon size used for the screening of *AmpC* β-lactamase [1] and ESBLs in members of Enterobacteriales [44].

PCR name	Targeted β-Lactamase(s)	Primer name	Primer sequence (5′ –3′)	Amplicon size (bp)
Simplex AmpC	*AmpC*	AmpC_for<br>AmpC_rev	TTCTATCAAMACTGGCARCC<br>CCYTTTTATGTACCCAYGA	550
Multiplex I TEM, SHV and OXA-1-like	TEM variants including TEM-1 and TEM-2	MultiTSO-T_for<br>MultiTSO-T_rev	CATTTCCGTGTCGCCCTTATTC<br>CGTTCATCCATAGTTGCCTGAC	800
	SHV variants including SHV-1	MultiTSO-S_for<br>MultiTSO-S_rev	AGCCGCTTGAGCAAATTAAAC<br>ATCCCGCAGATAAATCACCAC	713
	OXA-1, OXA-4 and OXA-30	MultiTSO-O_for<br>MultiTSO-O_rev	GGCACCAGATTCAACTTTCAAG<br>GACCCCAAGTTTCCTGTAAGTG	564
Multiplex II CTX-M group 1, group 2 and group 9	Variants of CTX-M group 1 including CTX-M-1, CTX-M-3 and CTX-M-15	MultiCTXMGp1_for<br>MultiCTXMGp1-2_rev	TTAGGAARTGTGCCGCTGYA[b]<br>CGATATCGTTGGTGGTRCCAT[b]	688
	variants of CTX-M group 2 including CTXM-2	MultiCTXMGp2_for<br>MultiCTXMGp1-2_rev	CGTTAACGGCACGATGAC<br>CGATATCGTTGGTGGTRCCAT[b]	404
	Variants of CTX-M group 9 including CTX-M-9 and CTX-M-14	MultiCTXMGp9_for<br>MultiCTXMGp9_rev	TCAAGCCTGCCGATCTGGT<br>TGATTCTCGCCGCTGAAG	561
CTX-M group 8/25	CTX-M-8, CTX-M-25, CTX-M-26 and CTX-M-39 to CTX-M-41	CTX-Mg8/25_for<br>CTX-Mg8/25_rev	AACRCRCAGACGCTCTAC[b]<br>TCGAGCCGGAASGTGTYAT[b]	326
Multiplex III ACC, FOX, MOX, DHA, CIT and EBC (plasmid-mediated AmpC)	ACC-1 and ACC-2	MultiCaseACC_for<br>MultiCaseACC_rev	CACCTCCAGCGACTTGTTAC<br>GTTAGCCAGCATCACGATCC	346
	FOX-1 to FOX-5	MultiCaseFOX_for<br>MultiCaseFOX_rev	CTACAGTGCGGGTGGTTT<br>CTATTTGCGGCCAGGTGA	162
	MOX-1, MOX-2, CMY-1, CMY-8 to CMY-11 and CMY-19	MultiCaseMOX_for<br>MultiCaseMOX_rev	GCAACAACGACAATCCATCCT<br>GGGATAGGCGTAACTCTCCCAA	895
	DHA-1 and DHA-2	MultiCaseDHA_for<br>MultiCaseDHA_rev	TGATGGCACAGCAGGATATTC<br>GCTTTGACTCTTTCGGTATTCG	997
	LAT-1 to LAT-3, BIL-1, CMY-2 to MY-7, CMY-12 to CMY-18 and CMY-21 to CMY-23	MultiCaseCIT_for<br>MultiCaseCIT_rev	CGAAGAGGCAATGACCAGAC<br>ACGGACAGGGTTAGGATAGY[b]	538
	ACT-1 and MIR-1	MultiCaseEBC_for<br>MultiCaseEBC_rev	CGGTAAAGCCGATGTTGCG<br>AGCCTAACCCCTGATACA	683

Multiplex IV VEB, PER and GES	GES-1 to GES-9 and GES-11	MultiGES_for	AGTCGGCTAGACCGGAAAG	399
		MultiGES_rev	TTTGTCCGTGCTCAGGAT	
	PER-1 and PER-3	MultiPER_for	GCTCCGATAATGAAAGCGT	520
		MultiPER_rev	TTCGGCTTGACTCGGCTGA	
	VEB-1 to VEB-6	MultiVEB_for	CATTTCCCGATGCAAAGCGT	648
		MultiVEB_rev	CGAAGTTTCTTTGGACTCTG	
Multiplex V GES and OXA-48-like	GES-1 to GES-9 and GES-11	MultiGES_for	AGTCGGCTAGACCGGAAAG	399
		MultiGES_rev	TTTGTCCGTGCTCAGGAT	
	OXA-48-like	MultiOXA-48_for	GCTTGATCGCCCTCGATT	281
		MultiOXA-48_rev	GATTTGCTCCGTGGCCGAAA	
Multiplex VI IMP, VIM and KPC	IMP variants except IMP-9, IMP-16, IMP-18, IMP-22 and IMP-25	MultiIMP_for	TTGACACTCCATTTACDG[b]	139
		MultiIMP_rev	GATYGAGAATTAAGCCACYCT[b]	
	VIM variants including VIM-1 and VIM-2	MultiVIM_for[c]	GATGGTGTTTGGTCGCATA	390
		MultiVIM_rev[c]	CGAATGCGCAGCACCAG	
	KPC-1 to KPC-5	MultiKPC_for	CATTCAAGGGCTTTCTTGCTGC	538
		MultiKPC_rev	ACGACGGCATAGTCATTTGC	

[a]Annealing position within the corresponding open reading frame (from the base A of start codon ATG).

[b]Y=T or C; R=A or G; S=G or C; D=A or G or T.

[c]This primer pair was previously described by [18].

References

1. Velusamy, S.; Gillespie, B. E.; Lewis, M. J.; Nguyen, L. T.; Headrick, S. I.; Schukken, Y. H.; Oliver, S. P. Phenotypic and genotypic antimicrobial resistance patterns of Escherichia coli isolated from dairy cows with mastitis. *Veterinary Microbiology* **2007**, *124*, 319–328, doi:10.1016/j.vetmic.2007.04.040.
2. Abouzeed, Y. M.; Hariharan, H.; Poppe, C.; Kibenge, F. S. B. Characterization of Salmonella isolates from beef cattle, broiler chickens and human sources on Prince Edward Island. *Comparative Immunology, Microbiology and Infectious Diseases* **2000**, *23*, 253–266, doi:10.1016/S0147-9571(99)00079-X.
3. Jawad, A. A.; Al-charrakh, A. H. Outer Membrane Protein C (ompC) Gene as the Target for Diagnosis of Salmonella Species Isolated from Human and Animal Sources. *Avicenna Journal of Medical Biotechnology* **2016**, *8*, 42–45.
4. Olsen, J. E.; Aabo, S.; Rasmussen, O. F.; Rossen, L. Oligonucleotide probes specific for the genus Salmonella and for Salm. typhimurium. *Letters in Applied Microbiology* **1995**, *20*, 160–163, doi:10.1111/j.1472-765X.1995.tb00416.x.
5. Akbari, M.; Bakhshi, B.; Peerayeh, S. N. Particular distribution of Enterobacter cloacae strains isolated from urinary tract infection within clonal complexes. *Iranian Biomedical Journal* **2016**, *20*, 49–55, doi:10.7508/ibj.2016.01.007.
6. Brisse, S.; Verhoef, J. Phylogenetic diversity of Klebsiella pneumoniae and Klebsiella oxytoca clinical isolates revealed by randomly amplified polymorphic DNA, gyrA and parC genes sequencing and automated ribotyping. *International Journal of Systematic and Evolutionary Microbiology* **2001**, *51*, 915–924.
7. Turton, J. F.; Perry, C.; Elgohari, S.; Hampton, C. V. PCR characterization and typing of Klebsiella pneumoniae using capsular type-specific, variable number tandem repeat and virulence gene targets. *Journal of Medical Microbiology* **2010**, *59*, 541–547, doi:10.1099/jmm.0.015198-0.
8. Kovtunovych, G.; Lytvynenko, T.; Negrutska, V.; Lar, O.; Brisse, S.; Kozyrovska, N. Identification of Klebsiella oxytoca using a specific PCR assay targeting the polygalacturonase pehX gene. *Research in Microbiology* **2003**, *154*, 587–592, doi:10.1016/S0923-2508(03)00148-7.

9. Mendez, B.; Tachibana, C.; Levy, S. B. Heterogeneity of tetracycline resistance determinants. *Plasmid* **1980**, *3*, 99–108, doi:10.1016/0147-619X(80)90101-8.
10. Marshall, B.; Tachibana, C.; Levy, S. B. Frequency of tetracycline resistance determinant classes among lactose-fermenting coliforms. *Antimicrobial agents and chemotherapy* **1983**, *24*, 835–40, doi:10.1128/AAC.24.6.835.
11. Marshall, B.; Morrissey, S.; Flynn, P.; Levy, S. B. A new tetracycline-resistance determinant, class E, isolated from Enterobacteriaceae. *Gene* **1986**, *50*, 111–117, doi:10.1016/0378-1119(86)90315-X.
12. Zhao, J.; Aoki, T. Nucleotide Sequence Analysis of the Class G Tetracycline Resistance Determinant from Vibrio anguillarum. *Microbiology and Immunology* **1992**, *36*, 1051–1060, doi:10.1111/j.1348-0421.1992.tb02109.x.
13. Warsa, U. C.; Nonoyama, M.; Ida, T.; Okamoto, R.; Okubo, T.; Shimauchi, C.; Kuga, A.; Inoue, M. Detection of tet(K) and tet(M) in Staphylococcus aureus of Asian countries by the polymerase chain reaction. *The Journal of antibiotics* **1996**, *49*, 1127–32, doi:10.7164/antibiotics.49.1127.
14. Burdett, V.; Inamine, J.; Rajagopalan, S. Heterogeneity of tetracycline resistance determinants in Streptococcus. *Journal of bacteriology* **1982**, *149*, 995–1004.
15. Kerrn, M. B.; Klemmensen, T.; Frimodt-Møller, N.; Espersen, F. Susceptibility of Danish Escherichia coli strains isolated from urinary tract infections and bacteraemia, and distribution of sul genes conferring sulphonamide resistance. *Journal of Antimicrobial Chemotherapy* **2002**, *50*, 513–516, doi:10.1093/jac/dkf164.
16. Post, V.; Hall, R. M. AbaR5, a large multiple-antibiotic resistance region found in Acinetobacter baumannii. *Antimicrobial agents and chemotherapy* **2009**, *53*, 2667–71, doi:10.1128/AAC.01407-08.
17. Maynard, C.; Bekal, S.; Sanschagrin, F.; Levesque, R. C.; Brousseau, R.; Masson, L.; Lariviere, S.; Harel, J. Heterogeneity among Virulence and Antimicrobial Resistance Gene Profiles of Extraintestinal Escherichia coli Isolates of Animal and Human Origin. *Journal of Clinical Microbiology* **2004**, *42*, 5444–5452.
18. Ellington, M. J.; Kistler, J.; Livermore, D. M.; Woodford, N. Multiplex PCR for rapid detection of genes encoding acquired metallo-beta-lactamases. *Journal of Antimicrobial Chemotherapy* **2007**, *59*, 321–322, doi:10.1093/jac/dkl481.
